# Determination of rivaroxaban in patient’s plasma samples by anti-Xa chromogenic test associated to High Performance Liquid Chromatography tandem Mass Spectrometry (HPLC-MS/MS)

**DOI:** 10.1371/journal.pone.0171272

**Published:** 2017-02-07

**Authors:** Priscilla Bento Matos Derogis, Livia Rentas Sanches, Valdir Fernandes de Aranda, Marjorie Paris Colombini, Cristóvão Luis Pitangueira Mangueira, Marcelo Katz, Adriana Caschera Leme Faulhaber, Claudio Ernesto Albers Mendes, Carlos Eduardo dos Santos Ferreira, Carolina Nunes França, João Carlos de Campos Guerra

**Affiliations:** 1 Hospital Israelita Albert Einstein, São Paulo, Brazil; 2 Santo Amaro University–UNISA, São Paulo, Brazil; Institut d'Investigacions Biomediques de Barcelona, SPAIN

## Abstract

Rivaroxaban is an oral direct factor Xa inhibitor, therapeutically indicated in the treatment of thromboembolic diseases. As other new oral anticoagulants, routine monitoring of rivaroxaban is not necessary, but important in some clinical circumstances. In our study a high-performance liquid chromatography-tandem mass spectrometry (HPLC-MS/MS) method was validated to measure rivaroxaban plasmatic concentration. Our method used a simple sample preparation, protein precipitation, and a fast chromatographic run. It was developed a precise and accurate method, with a linear range from 2 to 500 ng/mL, and a lower limit of quantification of 4 pg on column. The new method was compared to a reference method (anti-factor Xa activity) and both presented a good correlation (r = 0.98, p < 0.001). In addition, we validated hemolytic, icteric or lipemic plasma samples for rivaroxaban measurement by HPLC-MS/MS without interferences. The chromogenic and HPLC-MS/MS methods were highly correlated and should be used as clinical tools for drug monitoring. The method was applied successfully in a group of 49 real-life patients, which allowed an accurate determination of rivaroxaban in peak and trough levels.

## Introduction

Rivaroxaban (Xarelto, Janssen Pharmaceuticals, Titusville, New Jersey) is an oral anticoagulant that directly inhibits activated factor X (FXa). It is indicated for the prevention of thromboembolism in patients with atrial fibrillation [1], the treatment of deep-vein thrombosis (DVT) [2] and the prevention of recurrent DVT and pulmonary embolism (PE) following an acute DVT in adults [3, 4], in prevention of venous thromboembolism (VTE) in patients undergoing total knee or hip replacement surgery [5–7].

It is postulated that rivaroxaban has predictable pharmacokinetics and pharmacodynamics [3, 8, 9]. However, there is an increasing interest in rivaroxaban monitoring in special clinical situations [10, 11], such as: acute renal failure, bleeding complications or thrombosis during treatment, prior to urgent surgery, life-threatening bleeding, stroke, overdose and suspected accumulation of drug [9]. In addition, a recent study showed that rivaroxaban passes into human breast milk and its safety has not been determined [12]. Future works may recommend the rivaroxaban monitoring in pregnancy and lactation.

According to the *Scientific* and Standardization *Committee* (SSC), through its subcommittee on *Control* of *Anticoagulation*, of the International *Society* on *Thrombosis* and *Haemostasis*, rivaroxaban can be monitored by indirect methods as activated partial thromboplastin time (APTT) with reservation for low drug concentrations; prothrombin time (PT); and by anti-FXa assays [13]. However, there is a controversial about PT in the literature, some authors pointed out PT as a good method [13], but others like Lindhoff-Last, Samama [14], explain that PT is not a proper test for rivaroxaban measurement due to a discreet alteration in this parameter and also the dependency of thromboplastin reagent used. Gosselin, Adcock Funk [15] mentioned that drug activity measurement by chromogenic anti-Xa assay must be preferred against PT, APTT or dilute Russell viper venom time (DRVVT) because it is a more precise measurement and present lower bias.

High-performance liquid chromatography-tandem mass spectrometry (HPLC-MS/MS) methods for direct dosage of anticoagulants plasmatic concentration as rivaroxaban have been reported [16–18]. HPLC-MS/MS is known as a standard gold method due to its high specificity and sensitivity. The main concerns about HPLC-MS/MS use are related to method development/validation and the usual absence of commercial calibrators and controls. Therefore, our aim was to develop an HPLC-MS/MS assay for an accurate rivaroxaban concentration determination in plasma samples with a simple sample preparation step.

## Material and methods

### Ethics statement

Signed informed consent was obtained from each patient in accordance with ethical protocol regulations approved by local institutional review boards governing research involving human subjects under CAAE number: 43080215.9.0000.0071 (Albert Einstein Israeli Hospital Ethical Comitee).

### Population

Forty-eight patients treated with rivaroxaban 10, 15 or 20 mg were included in this study. Inclusion criteria for rivaroxaban patients were: over 18 years old, under rivaroxaban treatment (no particular indication was differentially pursued), and able to provide informed consent. Exclusion criteria for the present study were: pregnancy or age younger than 18 years of age.

Signed informed consent was obtained from each patient in accordance with ethical protocol regulations approved by local institutional review boards governing research involving human subjects under CAAE number: 43080215.9.0000.0071

### Chemicals

Rivaroxaban and its structurally analogous Internal Standard—IS (rivaroxaban-d4) were obtained from Santa Cruz Biotechnology (Santa Cruz, CA). Methanol in HPLC grade was obtained from JTBAKER (Avantor Performance Materials, Mexico, Mexico). Water was purified by the Milli Q system (Millipore Waters, Darmstadt, Germany). Blank plasma was obtained from healthy volunteers subjects. Formic acid was purchased from SIGMA ALDRICH (Sigma Aldrich, São Paulo, Brazil).

### Samples

Blood sample was collected into citrated plastic tubes (Sarstedt, Brazil), two hours before (trough) and two hours after drug intake (peak), centrifuged at 4500 rpm for 15 minutes at 10°C. Plasma was separated and stored below– 80°C until analysis.

Sample preparation was performed by protein precipitation with methanol. Two hundred microliters of plasma from calibration, quality control, or patient samples were placed to 7 mL plastic centrifuge tubes. Methanol (400 μL) containing deuterated IS (Rivaroxaban-d_4_, 500 ng/mL) was added to deproteinate samples. The samples were then centrifuged at 4000 rpm for 10 min at 4°C. The supernatants were filtered through a 0.22 μm PVDF filter (Millex^®^-GV, Merck Millipore, Cork, Ireland) and transferred to an amber clean autosampler vial with insert for analysis. Finally, 2 μL was injected into the HPLC-MS/MS system. For suppression ion study the Rivaroxaban-d_4_, 500 ng/mL, methanolic solution was replaced by methanol.

Rivaroxaban levels were determined by HPLC-MS/MS and by using an anti-factor Xa activity assay (STA^®^-Liquid Anti-Xa) at the Clinical Laboratory of Hospital Israelita Albert Einstein, Sao Paulo, Brazil.

### HPLC–MS/MS method

Chromatography was performed on an Agilent 1260 LC system (Agilent Technologies, Mississauga, Canada). The compounds were eluted from a Kinetex C18 HPLC column (100 × 3 mm, 2.6 μm particle size from Phenomenex, Torrance, CA) in an isocratic gradient consisting in 40% (A) ultrapure water containing 0.01% formic acid and 60% (B) methanol 0.01% formic acid as mobile phases. The flow rate and column temperature were set at 0.5 mL/min and 40°C, respectively. The LC system was coupled to an SCIEX QTRAP 5500 tandem mass spectrometer (SCIEX, Concord, Canada) fitted with electrospray ionization (ESI) source.

The operating parameters were ionspray voltage 5000 V, curtain gas 10 (arbitrary units), nebulizer gas 40 (arbitrary units), auxiliary gas 45 (arbitrary units) and probe temperature 600°C. The compounds were monitored in positive ion mode using multiple-reaction monitoring (MRM); the transitions, declustering potentials, collision energies and collision cell exit potentials are summarized in Table 1. For rivaroxaban two MRM transitions were used, one quantifier and one qualifier.

**Table 1:** Mass spectrometry parameters for rivaroxaban monitoring.

Analyte	MRM transitions	Type	RT (min)	DP (V)	EP (V)	CXP (V)
Rivaroxaban	435.9 → 144.9	Quantifier	1.8	156	10	18
	435.9 → 231.1	Qualifier	1.8	156	10	28
Rivaroxaban-D4	440.1 → 144.9	Internal Standard	1.8	116	10	18

MRM: multiple-reaction monitoring; RT: retention time; DP: declustering potential; EP: entrance potential; CXP: collision cell exit potential.

The software packages Analyst 1.6.2, PeakView 2.2 and MultiQuant 3.0 (SCIEX, Concord, Canada) were used, respectively, for mass spectral data acquisition and quantitation.

### HPLC–MS/MS validation

All validation steps were evaluated according to international guidelines [19–21].

### Selectivity

Assay selectivity was performed to evaluate any interference from endogenous matrix. Different lots of drug-free plasma samples (normal, hemolysate, lipemic and icteric) together with spiked plasma of rivaroxaban at lower limit of quantification level (LLOQ = 2 ng/mL) were processed and analyzed. The degree of co-eluting interferences was assessed by comparing the MRM chromatograms of drug-free plasmas with spiked plasma of analyte. The responses of interfering peaks in the retention time of the drug and the IS must be lower, respectively, than 20% and 5% of the response in the concentration employed. The samples indexes of lipemia, hemolysis and icterus were identified by visual inspection in +, ++ or +++, from the lowest to the highest. The samples were divided into ten groups based on sample type and index.

### Carry-over

Carry-over in general is serial in nature and is caused by residual analyte from a sample analyzed earlier in the run [22]. Carry-over was tested by injecting two extracted blank samples sequentially, immediately after an upper limit of quantification (ULOQ = 500 ng/mL) sample injection.

### Matrix effect

The interfering compounds present in matrix may strengthen or reduce the analyte detection of interest; this effect can lead sometimes to erroneous results.

Matrix effects were studied in two ways [23]: by injecting blank plasma samples onto the chromatographic column, during continuous infusion of rivaroxaban and rivaroxaban-d4 in the mass spectrometer; and, by comparison of the signal response of a standard present in a post-extraction spiked sample to the response of a standard in neat solution. The continuous infusion lead us qualitative information and the another method shows us the quantitative.

In the infusion method, it was used a 10 μL/min continuous post column infusion of a solution containing rivaroxaban (200 ng/mL) and rivaroxaban-d4 (200 ng/mL). During the infusion, samples prepared from drug free plasma without internal standard were subjected to LC–MS/MS analysis, and the MS/MS responses of the MRM transitions were monitored.

The effect of matrix type was investigated by the analysis of blank plasma of all groups cited in selectivity test, spiked, after protein precipitation, with rivaroxaban at two concentration levels (2.5 and 400 μg/mL) and rivaroxaban-d4 (67 ng/mL). These samples were compared with rivaroxaban spiked in water: methanol solution (1:2) containing the same concentrations.

The matrix effect was evaluated by calculating the matrix factor (MEF) for area response, using the following formula:

MEF=PlasmaanalyteareaPlasmaISareaAnalyteareainsolutionISareainsolution

The variability (relative standard deviation, RSD) of matrix effect at each concentration level should be less than 15%.

### Linearity

The linearity of the method was assessed by processing (in triplicate) a six-point calibration curve over the concentration range of 2–500 ng/mL on three consecutive batches. Calibration curves were built by fitting the analyte concentrations of the calibrators versus the peak area ratios of the analyte to IS using least-squares linear regression analysis with a weighting factor of 1/x^2^. LLOQ was defined as the lowest plasma concentration in the calibration curve.

Allowable dilutions that yield true results within and outside the measuring interval were evaluated. For this propose, a plasma sample containing rivaroxaban at 390 ng/mL was submitted to dilution in different proportions with blank plasma. The criteria for acceptability of the data included accuracy within 100 ± 15%.

### Recovery

The recovery of the analyte and the IS was determined by measuring the peak area responses from plasma samples spiked with analyte before extraction (in quintuplicate) with those from methanolic solution with same concentration. Analytical recoveries were calculated as the measured concentrations divided by the expected concentrations and expressed as a percentage. The recovery of rivaroxaban were evaluated in quintuplicate at three concentration levels of 10, 100 and 500 ng/mL from peak area ratios (rivaroxaban/IS) of assayed samples compared to the reference standard prepared in methanol. The recovery was calculated using the formula:

$$Recovery\;(\%)=\frac{Plasmatic\;concentration}{Concentration\;in\;solution}\;x\;100$$

### Accuracy and precision

The precision and accuracy were evaluated by analyzing quality controls (QCs) at plasma concentrations of two LLOQ, 2.5, 125, 400 and 800 ng/mL in five replicates on three separated days. The QC with 800 ng/mL was submitted to a prior dilution in blank plasma, to test accuracy dilution, so the concentration expected after extraction was 400 ng/mL. The acceptability criteria of the data included accuracy within 100 ± 15% of the nominal values, and precision within 15% RSD%, except for LLOQ at which both precision were within 20% and accuracy were within 100 ± 20%.

### Stability

Stability experiments were carried out to examine the analyte stability in plasma samples under different conditions. The stability assay was assessed using QC samples fortified with rivaroxaban at low and high concentrations (2.5 and 400 ng/mL). The storage conditions studied were as follows: at room temperature for 24 h, refrigeration at 4°C for 7 days, freeze at –20°C followed by thawing at room temperature (three freeze-thaw cycles), freeze at –20°C for one month, and freeze at -80°C for eight months. Analyses were performed in triplicate for each condition and level of concentration. The concentrations obtained in the QC samples after each storage condition were compared to those from freshly prepared QC samples. Acceptable results included a maximum concentration difference of 15% when comparing QC samples under storage with fresh QC samples.

### Anti-factor Xa activity assay

Rivaroxaban levels were determined using an anti-factor Xa activity assay (STA^®^-Liquid Anti-Xa) using STA^®^-Rivaroxaban Calibrator and STA^®^-Rivaroxaban Controls on the STA-R Evolution^®^ analyzer (Stago Diagnostica, Asnieres, France). External laboratory was used for a second measurement of anti-factor Xa activity assay.

### Data analysis

Statistical analyses were performed using GraphPad Prism version 5.00 (GraphPad Software, San Diego California, USA, www.graphpad.com) and SPSS software (SPSS for Windows, version 20.0, IBM Corp., Armonk, NY, USA). All statistical tests were performed at 5% significance.

The association between rivaroxaban anti-factor Xa activity and HPLC-MS/MS were determined by Spearman correlation. Additionally, the agreement between them is presented in a Bland Altman differences plot.

We evaluated the individual factors that could be influencing the plasmatic concentration results from HPLC-MS/MS method by using a multivariate generalized linear model regression (gamma -distributed error and log -link) adjusted for age, drug dosage, sex, body weight and serum creatinine level.

## Results

### High Performance Liquid Chromatography—tandem Mass Spectrometry (HPLC-MS/MS) validation

**Fig 1:**
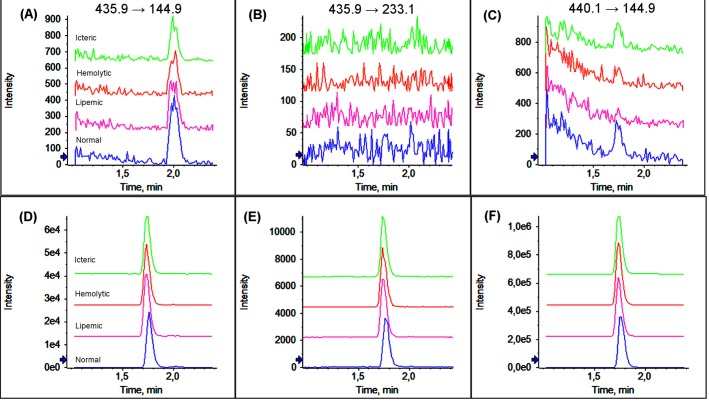
The matrix effect according to sample type. The chromatograms shows in blue, pink, red and green the multiple reaction monitoring (MRM) for normal, lipemic, hemolytic and icteric samples, respectively. Figs A, B and C shows the MRM transitions for rivaroxaban (435.9 → 144.9 and 435. 9 → 233.1) and for IS (440.1 → 144.9) in drug-free samples and D, E and F in samples spiked with rivaroxaban (400 ng/mL) and rivaroxaban-d4 (67 ng/mL).

According to European Medicines Agency, National Sanitary Vigilance Agency (ANVISA) and Food and Drug Administration (FDA), absence of interfering components is accepted when the response is less than 20% of the lower limit of quantification for the analyte, and 5% for the IS [19–21]. Consequently, thirty-two different plasma samples were analyzed for selectivity test (Table 2). Our results showed that interfering peaks did not exceed the allowable limit of 5% for IS retention time and were not observed interfering peaks at rivaroxaban retention time (Fig 1). So, the method is shown to be selective and carry-over effects between the analyses were not observed. The interfering peaks in the analyte retention time and IS, after ULOQ injection, did not exceed the permissible limits of 20 and 5% respectively.

**Table 2:** Number of samples according to sample type.

**Sample type**	**Number of samples**			
		Index		
		+	++	+++
**Normal**	7			
**Haemolysed**		3	2	4
Lipemic		3	3	2
Icteric		3	4	1

**Fig 2:**
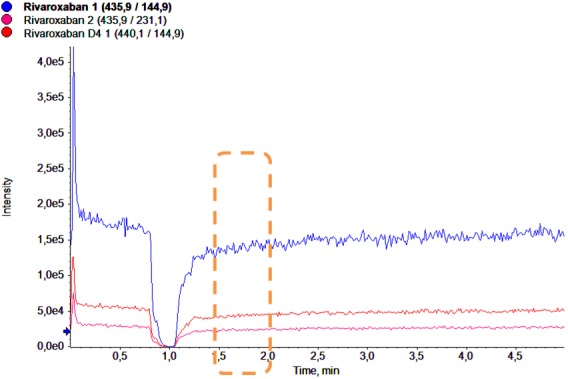
The matrix effect by infusion method. The chromatogram shows in blue, red and green the multiple reaction monitoring (MRM) for quantitative and qualitative detection of rivaroxaban and Internal Standard (IS), respectively. The orange dashed line highlight the rivaroxaban retention time expected.

The assessment of matrix effect by signal response ratio method showed that the ion suppression effects produced by endogenous matrix were in the range of acceptable limits (RSD < 15%). Matrix effects not interfere with the analyte signal in infusion method in expected retention time (Fig 2).

The calibration curves, ranging from 2–500 ng/mL, were constructed in triplicate on three different days by plotting the peak area ratio (analyte/IS) of plasma standard versus the nominal concentration. The best linear fit, higher accuracy and lowest bias was achieved using a regression analysis weighing 1/X^2^ factor. The linear regression equation was y = 0.0029x + 0.0007 with correlation coefficient greater than 0.995 (r^2^ = 0.9996).

The mean recovery of plasma samples from low to high concentrations after treatment was 95.2 ± 7.2% for 10 ng/mL, 97.4 ± 3.1% for 100 ng/mL, and 101.0 ± 1.5% (500 ng/mL) for rivaroxaban. These results suggest that there were no relevant differences in plasma treatment recoveries.

Intra and inter-day precision and accuracy outcomes of QC samples are shown in Table 3 and were all below 15% and within 100 ± 15%, respectively, confirming the method accuracy and precision.

**Table 3:** Intra-day and inter-day precision and accuracy values of plasmatic rivaroxaban.

QC(concentration)	Intraday 1 (n = 5)		Intraday 2 (n = 5)		Intraday 3 (n = 5)		Inter-day (n = 15)	
	Accuracy (%)	Precision (CV %)	Accuracy (%)	Precision (CV %)	Accuracy (%)	Precision (CV %)	Accuracy (%)	Precision (CV %)
LLOQ (2.0 ng/mL)	97.7	3.8	95.4	5.8	101.8	3.9	98.3	5.1
Low (2.5 ng/mL)	101.1	4.9	98.7	3.9	104.9	1.7	101.6	4.3
Medium (125 ng/mL)	91.8	7.1	93.3	2.2	100.3	1.4	95.1	5.6
High(400 ng/mL)	91.5	12.9	89.9	6.4	98.6	2.9	93.3	8.8
Dilution (400 ng/mL)	96.9	1.3	95.5	3.0	100.9	4.0	97.8	3.7

QC: quality control; LLOQ: lower limit of quantification level.

The subject samples to the different stability conditions were analyzed against the freshly made QC samples. The mean values and standard deviations (SD) of the ratios between the concentrations found and initial concentration were used to calculate accuracy and precision. The results were summarized in Table 4 and indicated that the analytes exhibit acceptable stability under those conditions with accuracy ranging from 99.2 to 110.3%.

**Table 4:** Stability data of rivaroxaban in human plasma.

Stability Nominal	Nominal concentration (ng/mL) (n = 3)	Accuracy (%)	Precision (% CV)
Freeze thaw (3 cycle)	2.5	104.0	4.8
	400	101.5	4.0
Bench top stability (24h. room temperature)	2.5	99.2	1.5
	400	99.4	3.3
In Injector (24h)	2.5	99.2	5.2
	400	99.4	0.6
In Injector (48h)	2.5	105.0	1.7
	400	102.7	0.8
7 days storage stability (4°C)	2.5	104.1	7.9
	400	101.0	1.5
30 days storage stability (-20°C)	2.5	104.0	7.0
	400	106.0	0.8
8 months storage stability (-80°C)	2.5	87.9	11.5
	400	110.3	7.6

### HPLC-MS/MS comparison to anti-factor Xa activity assay

**Fig 3:**
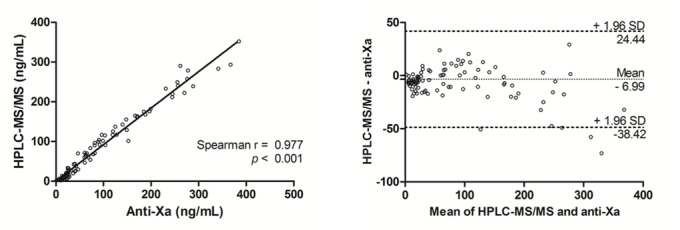
Method comparison HPLC-MS/MS assay and the anti-Xa assay (n = 96): (A) Spearman correlation of rivaroxaban results obtained by the HPLC-MS/MS assay and the anti-Xa assay used for rivaroxaban measurement from STAGO performed on the STA-R Evolution^®^ analyzer; (B) Bland-Altman analyses.

**Fig 4:**
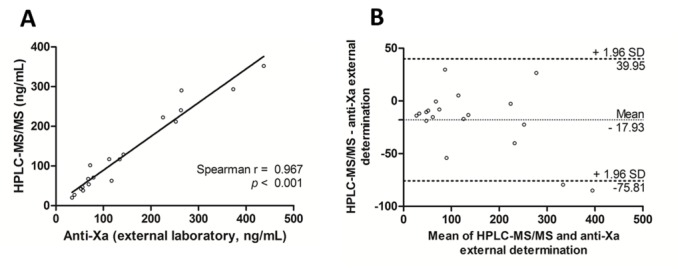
Method comparison HPLC-MS/MS assay and the anti-Xa assay from external laboratory (n = 19): (A) Spearman correlation of rivaroxaban results obtained by the HPLC-MS/MS assay and the anti-Xa assay used for rivaroxaban measurement from external laboratory; (B) Bland-Altman analyses.

Ninety-six samples were analyzed by HPLC-MS/MS and by STAGO anti-factor Xa activity. Additionally, twenty-one of them were also analyzed by anti-factor Xa assay in an external laboratory. Two samples used in external comparison were excluded because their values were lower than LLOQ. Figs 3 and 4 show the strong correlation between HPLC-MS/MS and different anti-Xa activity assays (r = 0.977, p < 0.001 and r = 0.967, p < 0.001). Aside these good correlations, the Bland-Altman analysis showed a mean difference of -6.9 ng/mL for plasma concentrations estimated by the anti-Xa assay (internal determination) in comparison with the HPLC-MS/MS (SD: 16 ng/ml; 95% limits of agreement: -38 to 24 ng/mL). If we compare with the external determination the mean difference was -17.9 ng/mL (SD: 30 ng/mL; 95% limits of agreement: -76 to 40 ng/mL). Higher concentrations showed higher deviations.

**Fig 5:**
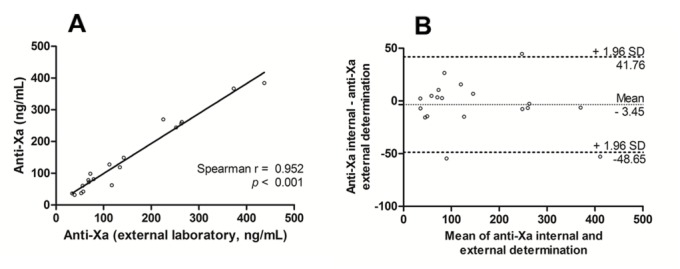
Interlaboratorial anti-Xa method comparison (n = 19). (A) Interlaboratorial comparison of rivaroxaban results obtained by anti-Xa activity assays; (B) Bland-Altman analyses.

As expected, the Spearman correlation between the same types of measurement was good (r = 0.952, p < 0,001, Fig 5) with a mean difference of -3.4 ng/mL, according to Bland-Altman analysis. The results showed a good agreement between the rivaroxaban plasmatic concentration measurements.

### Method application

The HPLC-MS/MS described and validated assay has been applied to plasma obtained before and after drug administration in 49 patients treated with rivaroxaban. According to rivaroxaban dosage (10, 15 and 20 mg), the peak means (± SD) plasmatic concentrations were 110 ± 95, 141 ± 92 and 183 ± 82 ng/mL, and the trough concentrations were: 21 ± 30, 40 ± 52 and 73 ± 103 ng/mL, respectively. As expected, it was possible to differentiate peak and trough plasmatic levels using this validated method.

Additionally, using the multivariate analyses we observed that the main factor influencing the plasmatic concentration in our group of patients is the drug dosage. This dependence to drug dosage is prevalent in peak measurements. It was observed a log increment of 0.863 (p = 0.002) for 15 mg and 1.142 (p < 0.001) for 20 mg, using 10 mg as reference.

## Discussion

HPLC-MS/MS is becoming an important tool for clinical laboratories because it is a very specific, selective and sensitive technique. Additionally, permits a wide range of applications and it is possible to obtain a large number of quantitative or qualitative results [24, 25]. Diverse compounds, as anticoagulant agents, can be directly measured by an HPLC-MS/MS method. This technique has much better selectivity than coagulation activity-based assays, enabling specific detection and quantification of coagulation inhibitors [18].

In this study, a HPLC-MS/MS method was developed and validated for rivaroxaban quantification using a simple sample preparation and chromatographic condition. Our method was shown to be precise, accurate, sensitive, specific and robust. Table 5 summarize the most important differences between the present method and previously published.

**Table 5:** Rivaroxaban HPLC-MS/MS method comparison.

Sample	Sample type	Sample preparation	LC column and Mobile Phase	LC-MS/MS analysis time	LLOQ	Linearity (ng/mL)	Stability	Ref.
Whole blood (citrate and heparin)Plasma (citrate and heparin)	No data	PP (MeOH)	C18MeCN and ammonium acetate buffer	6 min	0.5 ng/mL (20 pg on column)	0.5–500	Room temperature≤ 8°C≤ 15°CFreeze thaw	[16]
Plasma (citrate)	No data	PP (MeOH/HCl)	BEH Phenyl Water, methanol, formic acid and ammonium acetate	2.5 min	0.5 ng/mL(0.5 pg on column)	0.8–800	Room temperature 4°C—20°C	[17]
Plasma (citrate)	No data	PP (MeCN)	BEH C8MeCN and ammonium acetate buffer	4.75 min	No data	23–750	Room temperature 4°C Freeze thaw -80°C	[18]
Plasma (EDTA)	No data	PP (MeCN) and Cyclone-C18-P-XL TurboFlow column	Phenyl Hexyl MeOH, MeCN and ammonium acetate buffer	6 min	1 ng/mL(100 pg on column)	1–500	Room temperature 4°C Freeze thaw	[26]
Plasma (citrate)	No data	Dilution with a basic solution, incubation for 2 hours and turbulent flow liquid chromatography	No data No data	No data	No data	5–1000	No data	[27]
Plasma (heparin)	No data	PP (MeOH/HCl)	BEH C18MeCN, ammonium acetate buffer and formic acid	No data	2.5 ng/mL (37.5 pg on column)	2.5–500	Room temperature 4°C- 20°C	[28]
Plasma (EDTA)	No data	PP (MeOH/formic acid)	BEH C18MeCN, ammonium acetate buffer	1.5 min	0.57 ng/mL (2.85 pg on column)	0.57–625	Room temperature Freeze thaw Injector -80°C	[29]
Plasma citrate	NormalIctericHemolyticLipemic	PP (MeOH)	C18MeOH, water and formic acid	5 min	2 ng/mL(4 pg on column)	0.5–500	Room temperature4°CFreeze thaw Injector -20°C-80°C	This work

LLOQ: lower limit of quantification; Ref.: references; PP: protein precipitation; MeCN: acetonitrile; MeOH: methanol; HCl: hydrochloric acid.

Our method took less than 20 min in sample preparation step. While, the method described by Rathbun, Tafur [27] used a longer sample processing period (more than two hours). Korostelev, Bihan [28] developed a HPLC–MS/MS method to quantify dabigatran and rivaroxaban, the study was conducted using plasma with a linearity range from 2.5 to 500 ng/mL with a LLOQ of 37.5 pg on column. Rohde [16] developed a rivaroxaban HPLC-MS/MS method linear from 0.50 to 500 ng/mL with a LLOQ of 20 pg on column. Our method was linear from 2 to 500 ng/mL and presented a LLOQ of 4 pg on column.

Diverse studies showed rivaroxaban stability in plasma at room temperature, + 4°C and—20°C [16, 17, 28]. Besides, Iqbal group [29] tested rivaroxaban at -80°C for only 60 days. Our study supports the rivaroxaban stability described above and additionally showed that rivaroxaban is stable at -80°C at least for eight months, with a good accuracy and precision.

Harenberg and collaborators [30] measured rivaroxaban, dabigatran and apixaban in plasma, serum and urine using a Point-of-Care Testing (POCT) assays for factor Xa- and thrombin inhibitors. Wiesen, Blaich [12] detected rivaroxaban in breast milk.

There is a considerable range of biological samples that can be used for rivaroxaban measurement. However, it is well described in literature that mass spectrometry based methods can suffer interference of biological matrix compounds. Consequently, as cited by Van Eeckhaut, Lanckmans [23], matrix effect evaluation should be a mandatory part of the validation procedure.

Rivaroxaban was measured by HPLC-MS/MS in whole blood, plasma citrate, plasma heparin and urine [16–18, 26]. The matrix effect tested by the infusion method was absent or presented no significant influence in plasma citrate as described by Kuhn, Gripp [17]and Schmitz, Boonen [18].

Previews studies have shown, in matrix effect tested by signal response comparison, non-relevant influence in plasma EDTA [29], whole blood, plasma citrate and heparin [16]. All cited articles assessed matrix effects only in normal samples. In the present work it was demonstrated not only non-significant interference for plasma citrate in normal samples, but also in icteric, lipemic and hemolytic samples. Additionally, it is important to note that the opacity of plasma samples is a relevant limitation for chromogenic assays [31]. So, this may be one of the reasons for the increase in development of HPLC-MS/MS methods for rivaroxaban measurement. Of course, there is limitation in HPLC-MS/MS technology, like high instrument cost and the need of expertise [16], but the good results are favoring this technology.

Rivaroxaban is absorbed rapidly, with maximum plasma concentration occurring 2–4 hours after drug administration [9]. The elimination of rivaroxaban from plasma occurs within a 5–13 hours depending on patient age [32, 33]. In our study, we have assessed rivaroxaban plasmatic concentration two hours before (trough) and two hours after (peak) drug administration, in patients receiving rivaroxaban in once daily regimen.

The chromogenic anti-FXa results were compared to measurements made by HPLC-MS/MS. Both methods showed a high degree of correlation, as cited in literature [3, 17, 34]. Higher concentrations showed higher deviations, this is may be justified by a lower number of samples with higher concentration. The higher bias in higher concentration it was also shown by Douxfils, Tamigniau [3]. Additionally, Mani, Rohde [34] recommended for an accurate determination of rivaroxaban levels the use of two different calibrator sets, one for high concentration and the other for low concentrations.

The results obtained in our study for the mean peak and trough levels were similar to those described by Mueck, Borris [35]. Samama, Contant [9] resumed rivaroxaban peak and trough plasmatic concentrations in different clinical settings. The results obtained in the present study are compatible in the trough dosage but are lower in peak levels. The same phenomenon was observed comparing our results with those cited by Baglin [1] and by Mueck, Schwers [36]. Bardy, Fischer [37] showed that chromogenic values overestimated HPLC-MS/MS values despite the correlation between the two methods and linear regression were highly significant. This may be a reason for the differences found, but not the only one. Further, clinical studies will be conducted to understand the others factors involved in our findings.

## Conclusion

In the present work it was developed and validated a fast, accurate and precise HPLC-MS/MS method for rivaroxaban determination. A simple sample preparation and a relative short chromatographic run time were used. The method was successfully applied to determine rivaroxaban concentrations two hours before and two hours after drug intake. Hemolytic, icteric or lipemic plasma samples can be used in rivaroxaban measurement by HPLC-MS/MS without interferences. The chromogenic and HPLC-MS/MS methods were highly correlated and should be used as clinical tools for drug monitoring.
